# To what extent do primary care practice nurses act as case managers lifestyle counselling regarding weight management? A systematic review

**DOI:** 10.1186/s12875-014-0197-2

**Published:** 2014-12-10

**Authors:** Sonja ME van Dillen, Gerrit J Hiddink

**Affiliations:** Strategic Communication, Section Communication, Philosophy and Technology, Centre for Integrative Development (CPT-CID), Wageningen University, P.O. Box 8130, 6700 EW Wageningen, the Netherlands

**Keywords:** Primary care, Systematic review, Obesity, Nutrition education, Communication

## Abstract

**Background:**

In this review study, we are the first to explore whether the practice nurse (PN) can act as case manager lifestyle counselling regarding weight management in primary care.

**Methods:**

Multiple electronic databases (MEDLINE, PsycINFO) were searched to identify relevant literature after 1995. Forty-five studies fulfilled the inclusion criteria. In addition, all studies were judged on ten quality criteria by two independent reviewers.

**Results:**

Especially in the last three years, many studies have been published. The majority of the studies were positive about PNs’ actual role in primary care. However, several studies dealt with competency issues, including disagreement on respective roles. Thirteen studies were perceived as high quality. Only few studies had a representative sample. PNs’ role in chronic disease management is spreading increasingly into lifestyle counselling. Although PNs have more time to provide lifestyle counselling than general practitioners (GPs), lack of time still remains a barrier. In some countries, PNs were rather ambiguous about their role, and they did not agree with GPs on this.

**Conclusion:**

The PN can play the role of case manager lifestyle counselling regarding weight management in primary care in the UK, and wherever PNs are working under supervision of a GP and a primary health care team is already developed with agreement on roles. In countries in which a primary health care team is still in development and there is no agreement on respective roles, such as the USA, it is still the question whether the PN can play the case manager role.

**Electronic supplementary material:**

The online version of this article (doi:10.1186/s12875-014-0197-2) contains supplementary material, which is available to authorized users.

## Background

In recent years, general practitioners (GPs) have faced a heavy workload. This led to a call for more practical support. The emergence of primary care practice nurses (PNs) came in a period of increasing awareness of chronic disease management [1]. In the UK, chronic disease services have shifted from secondary care to general practice and from GPs to practice nurses (PNs). A new UK GP contract requires adherence to chronic disease management tools, and facilitating self-management is recognized as an important component [2]. In the Netherlands, PNs are specially trained to be employed in GP practices: currently three quarters of all practices have a PN at their disposal [1]. Besides chronic disease management PNs provide an increasing proportion of preventive lifestyle advice. PNs work under supervision of GPs, which means that PNs cannot refer patients or prescribe medicines without permission of a GP. In the UK, the Netherlands, Sweden, Finland, Australia and New Zealand, such a collaborative system has been implemented [3]. A review revealed that lifestyle advice provided by GPs was rather general [4]. Moreover, research showed that GPs perceive barriers in lifestyle counselling [5]. To overcome these barriers, PNs can partially take over lifestyle counselling [6]. There is evidence that supports effectiveness of lifestyle interventions delivered by PNs to affect positive changes on outcomes associated with prevention of chronic disease, including weight and dietary and physical activity behaviors [7]. Moreover, a Cochrane review suggests that appropriately trained PNs can produce as high quality care as GPs and achieve as good health outcomes for patients [8].

The obesity epidemic is escalating and creating enormous disease burdens. Nearly 1.5 billion adults were overweight in 2008, and of these half a billion were clinically obese – almost double the rates of 1980 [9]. A large proportion will need help with weight management: the primary health care team (such as GPs and PNs) have an important role in the identification, assessment and management of overweight and obese adults and children [10]. GPs and PNs should advise patients on weight, diet, and physical activity to motivate patients to change. However, it is not known what is PNs’ actual role in lifestyle counselling in primary care at present. A clear overview of studies performed in the field is missing. A solid description of the current situation in terms of PNs’ attitudes and their perceived barriers, as well as their lifestyle counselling practices is needed.

Furthermore, PNs have to work together with other health professionals in primary care and public health, such as GPs, practice assistants, and dieticians. In an interdisciplinary model, different members of a primary health care team take appropriate and complementary roles to increase treatment effectiveness and health care system efficiency, as well as coordination with resources outside primary care setting [11]. In the Netherlands, the core primary health care team consists of a GP, PN and practice assistant. The practice assistant is responsible for administrative duties, while the PN supports the GP in the care for chronically ill people [1]. PNs are actually generalists, while competent in several different fields, but in integrated care they might cooperate with other professionals, such as dieticians who are specialized in the area of nutrition. Assessment of the contribution that various health professionals in primary care can make is relevant to explicating role responsibilities. Understanding of collaborative practice is needed, as well as comparison of the outcomes of their individual lifestyle counseling practices in terms of patients’ satisfaction, lifestyle behavior and health. In some countries, shift in extended care of PNs might cause tension. For example, GPs and PNs in the USA do not agree about their respective roles in delivery of primary care [12]. In other countries, such as the Netherlands, it runs relatively smoothly.

It is important that one professional has the lead and overview; this is indicated as case management. Case managers manage collaborative process of assessment, planning, facilitation and advocacy for options and services to meet individual's health needs through communication and available resources to promote quality cost-effective outcomes [13]. Coordination of care, patient education and counselling, and monitoring of health outcomes, are all integral part of nurse case management. For example, Dutch lifestyle interventions in primary care were primarily undertaken by case managers like lifestyle advisors or PNs under supervision of GPs [14,15]. Other potential case managers are GPs themselves, dieticians, diabetes educators, exercise specialists, and psychologists. We wonder whether the PN can play the role of case manager lifestyle counselling regarding weight management in primary care. To our knowledge, no systematic review has been performed in this field before. Since we are specifically interested in the case manager role, we will not take into account any regulatory issues as well as patients’ perceptions. However in case of intervention studies, we are interested in the lifestyle behavior and health outcomes for patients.

Therefore, the aim of this review is to describe PNs’ actual role in lifestyle counselling in primary care and their cooperation with other health professionals.

The underlying research questions are:

- What is known about the main outcomes of studies conducted regarding PNs’ actual role in lifestyle counselling in primary care?
- What is known about how PNs’ role in lifestyle counselling relates to the role of other health professionals in primary care and public health?

This review will provide an overview of the state of the art with respect to PNs’ involvement in lifestyle counseling in primary care, and we will use this as a first approach to discuss for the near future whether the PN can act as case manager lifestyle counselling regarding weight management in primary care.

## Methods

### Search strategy

A computerized literature search of multiple electronic databases was performed (MEDLINE, PsycINFO) with EBSCO-host as resource for relevant papers published between 1 January 1995 and 1 July 2013, using the following keywords as search strategy in the title or abstract (Table 1). The year 1995 was chosen, because PNs were introduced into primary health care in the nineties. This review is a first approach for carefully examining the role of PN as case manager lifestyle counselling regarding weight management, based on an overview about the description of the actual role of PNs in lifestyle counselling and its cooperation with other health professionals. We also checked Cochrane library for relevant reviews.

**Table 1:** **Search strategy of electronic databases MEDLINE and PsycINFO with EBSCO-host**

**Search strategy**	
Step 1	Practice nurse OR nurse practitioner OR primary nursing care OR primary care nurse OR primary health care nurse OR PHC nurses
Step 2	Guidance OR counselling OR communication OR advice OR health education OR health promotion OR prevention OR lifestyle behaviour OR chronic disease OR chronic illness
Step 3	Nutrition OR diet OR food OR physical activity OR exercise OR physical fitness OR weight OR overweight OR obesity OR adiposity OR corpulence
Step 4	Role OR position OR task OR duty OR responsibility OR competency OR skill OR expertise OR mission OR profession OR contribution OR lifestyle advisor OR case manager OR case management
Step 5	Cooperation OR collaboration OR teamwork OR alliance OR referral OR interdisciplinary OR multidisciplinary OR network OR delegation
Combine search 1, 2 and 3	
Combine search 1, 2 and 4	
Combine search 1, 2 and 5	

### Inclusion criteria

Studies had to be peer-reviewed journal articles, which addressed PNs’ involvement in lifestyle counselling in primary care. Only studies published in English language and original papers were included, of which full text was available. The review excluded studies that were based in hospitals, studies among children, and studies about patients’ perceptions.

### Study selection

**Figure 1:**
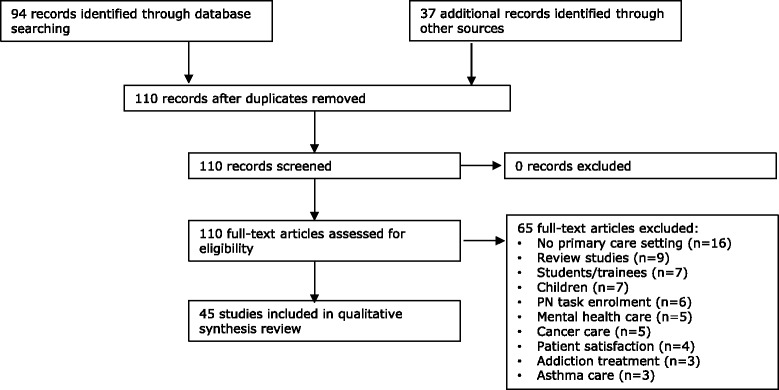
**Flow chart for study selection.**

After the electronic database search by one reviewer (SvD), reference lists of articles and reviews were screened for other potentially relevant papers. Figure 1 shows the flow chart for study selection.

### Data extraction

Data extraction of main characteristics was performed, namely:

- Author, year of publication, country;
- Study design: cross-sectional/longitudinal; randomized controlled trial (RCT)/interview/questionnaire;
- Sample: random sampling, number of PNs or GPs;
- Main outcomes for studies about PNs’ actual role in lifestyle counselling in primary care;
- Main outcomes for studies about PNs’ cooperation in the field of lifestyle counselling with other health professionals in primary care and public health.

### Assessment of study quality

Two reviewers (SvD and GH) independently assessed study quality of selected studies. The studies were judged on the following quality criteria, which were applied before in a review study among GPs [4]:

- Clear description of study aim (e.g. consistency in research questions, measurement instrument, results and conclusions);
- Appropriate size of study population (e.g. report of rationale for sample size);
- Sound selection of study population (e.g. random, stratified);
- Representative sample (e.g. no over-representation of female PNs, no over-representation of older PNs);
- Good response rate (e.g. > = 80% for phone or face-to-face interviews, > = 50% for mail questionnaires or classroom papers, > = 30% for Internet questionnaires) or low refusal rate/drop-out;
- Efforts were undertaken to optimize response rate (e.g. personalized letters, postage paid return envelope, reminders, incentive/gifts, simple and short measurement instrument, inclusion of group new respondents);
- Measurement instrument was well-developed (e.g. based on validated measures, prior research or reviewed literature);
- Measurement instrument was tested before use (e.g. pilot-test, pre-test for clarity, test-retest);
- Appropriate measurement instrument (e.g. distinguishable answer categories, Likert scales);
- Suitable report of study limitations and shortcomings (e.g. to overcome bias).

A maximum score of ten plusses could be achieved. Studies with eight plusses or more were seen as high quality. Studies with five, six or seven plusses were considered as medium quality studies, and studies with four or less plusses were considered as low quality studies. Although arbitrary, this quality assessment was done by two independent researchers, who compared results and reached consensus.

### Conditions for acting as case manager in primary care

The results were viewed against conditions for acting as case manager lifestyle counselling regarding weight management in primary care [15]:

- Time;
- Money;
- Interest;
- Perceived expertise;
- Competences;
- Type of intervention activity

## Results and discussion

### Main characteristics of studies

An additional file shows the main characteristics in more detail [see Additional file 1]. Thirty-three studies were performed in Europe, specifically in the UK (18 studies) and the Netherlands (10 studies). Six studies were done in Australia and five studies in the USA.

Our review delivered a wealth of studies (45 in total), which all concerned cross-sectional studies. The cross-sectional study of Philips et al. (2009) [16] was also accompanied by a longitudinal study. Both qualitative and quantitative studies were found, with slightly more quantitative studies. Twelve RCTs were found and four national surveys. Next to mail questionnaires, we also found observations, focus groups, and interviews. This order of scientific rigidity will be followed in the evaluation and discussion of the results.

Samples ranged between 1 [17] and 606 PNs [18]. Most studies were published after 2000, especially in the last three years.

### Main outcomes for studies about PNs’ actual role in lifestyle counselling in primary care

All 45 studies found were indeed discussing PNs’ actual role in lifestyle counselling in primary care. Outcomes of these studies were classified into three categories: positive, neutral, and negative. In order to be evaluated as “positive”, the study should show that PNs’ role in lifestyle counselling is warranted. An additional file shows in more detail that the majority of studies were positive about PN’s actual role in lifestyle counselling in primary care [see Additional file 1]. Twenty-six studies demonstrated that PNs’ role in lifestyle counselling is warranted. Furthermore, 10 out of 12 RCTs with an intervention by PNs resulted in positive outcomes. Six intervention studies in the UK and the Netherlands showed that PNs can achieve equally good health outcomes as GPs for different kinds of diseases [19-24]. More intensive interventions initiated the most changes [25]. Three other intervention studies revealed that consultations with PNs were significantly longer than those of GPs, and patients were significantly more satisfied with PNs’ care [26-28]. Two surveys showed that British PNs reported high levels of physical activity counselling [29,30] and three other surveys revealed that physical activity counselling increased in recent years in the USA [18,31,32]. Two observational studies comparing PNs’ and GPs’ counselling practices showed that Finnish and Dutch PNs more often discussed diet and physical activity than GPs [33,34]. In the UK, PNs and GPs agreed that PNs have main responsibility to cardiovascular health promotion [35], and weight management was considered as just one aspect of the busy and diverse role of the PN [36]. Australian PNs performed at least six roles, often alternating rapidly between them [16].

However, eight studies were neutral about PNs’ actual role in lifestyle counseling in primary care. According to these studies, parts of the role of PNs in lifestyle counselling in primary care are debatable. A survey among PNs and GPs in Ireland showed that there is some congruence in opinion regarding current role of the PN [37]. Although British PNs were more likely to raise weight issues than GPs, only 9% self-reported to present solutions or discuss health promotion [38], and weight management seems to be based on brief opportunistic intervention undertaken mainly by PNs [39]. Interviews with American PNs identified that realities of PN practice were often different from idealized practice, role identity was considered as ambiguous, and a need to blend medical and nursing models [40].

Moreover, eleven studies were negative about PNs’ actual role in lifestyle counseling in primary care. These studies showed that PNs’ role in lifestyle counselling in primary care seems to be limited. Two RCTs found no outcomes of a intervention by PNs. A RCT showed that adding nurse practitioners (NPs) to Dutch general practice teams did not reduce GPs’ workload, implying that NPs are used as supplements, rather than substitutes [41]. Another RCT showed that follow-up by PNs in the UK does not necessarily translate into better care or clinical outcomes [42]. Results from a national survey in Australia showed that PNs are a clinically experienced workforce whose skills are not optimally harnessed to improve care of growing number of people with chronic conditions [43]. Another national survey among American GPs and PNs indicated that they do not agree about their respective roles in delivery of primary care for complex chronic conditions [12]. The majority of Australian PNs agreed in a survey that PNs’ role could be expanded to include autonomous functioning, while most GPs were amenable to some extension of nursing practice [44]. Regarding weight management, PNs in the UK self-reported that they mainly offer general nutrition and exercise advice [45], and GPs and PNs in another British survey showed diversity with respect to their own relative importance in weight management [46].

### Main outcomes for studies about PNs’ cooperation in the field of lifestyle counselling with other health professionals in primary care and public health

Fourteen studies were found to answer the second research question. In all these studies, the relationship with GPs was described. In six studies, it was explicitly mentioned that PNs worked under supervision of a GP [16,22,23,34,44,47]. In a Dutch intervention study for example, NPs intensively guided behavioral change process, while GPs oversaw patients’ progress [22]. A national survey showed that for all referrals made to medical specialists, a GP was involved in the nurse-patient encounter [47]. They concluded that greater flexibility in PNs’ role will maximize efficient use of nurses’ skills in primary health care context in Australia. In Ireland, over 85% of PNs and GPs surveyed appear to have an agenda in chronic disease management, and strong primary care teams are under development [37]. Most GPs in the UK seem to refer obese patients to their PN instead of using external sources of support [39]. NPs interviewed in Canada described their expectation in collegial partnerships with GPs, but in reality they work in more traditional hierarchical relationships [48]. Besides GPs, cooperation with dieticians was discovered in eight studies [22,23,34,36,39,46,49,50]. Collaboration with several disciplines in general practice, like dietician, was perceived as an important facilitator in interviews with Dutch PNs and GPs [49]. Australian PNs were reluctant to cross professional boundaries: they often refer to ‘‘not being a dietician”, when explaining the scope of their own practice [50]. Another collaboration partner appeared to be the practice assistant (two studies). An observational study in Australia showed that PNs spent 45% of their time in contact with patients and 16% in contact with other general practice staff, including bridging the gap between clinical and administrative staff [16]. Interviewed PNs in the UK felt that health care assistants encroached their territory [51].

### Study quality

Studies in this review were assigned six plusses on average. Thirteen studies were perceived as high quality, 20 studies were considered as medium quality studies, and 12 studies were judged as low quality.

All studies had a clear description of study aim, and the majority had a well-developed or appropriate measurement instrument, suitable report of limitations, and an appropriate size of study population. Less than half of the studies had a good response rate, took efforts to optimize response rate, tested the measurement instrument before use, or had a sound selection of study population. Only few studies were representative.

All 45 papers discussed PNs’ actual role in lifestyle counselling in primary care, and addressed the first research question. Of these, 26 were positive, 8 were neutral, and 11 were negative. The 26 studies considered as positive, were of high or medium quality. Not all papers addressed PNs’ cooperation with other health professionals. Fourteen studies were found for answering the second research question. The studies found were of medium quality.

### Analysis on the basis of conditions for acting as case manager in primary care

No study explicitly discussed PNs’ role as case manager in primary care. Conditions for acting as case manager in primary care were found in 27 studies.

With respect to the condition time (in relation to task), four studies showed that PNs have more time to provide lifestyle counselling than GPs [23,33,34,39] and that frequency of PNs’ lifestyle counselling has increased in recent years [32]. However, six studies discussed time as a barrier for providing lifestyle counselling, of which five lack of time [40,50,52-54], but the majority of PNs in the study of Steptoe et al. (1999) [35] disagreed with the statement that they had no time for health promotion.

Regarding money, six studies identified funding as barrier for lifestyle counselling [18,31,32,40,43,52]. According to PNs in the study of Donelan et al. (2013) [12], they should be paid equally for the same clinical service. However, PNs did not rank adequate reimbursement as being as important as GPs in influencing their decision to counsel [54].

According to the factor interest, PNs were generally positive towards lifestyle counselling. However, motivation of PNs to continue implementation of a Dutch lifestyle intervention was lower compared with GPs or physiotherapists [55].

Regarding expertise, six studies were found. Grimstvedt et al. (2012) [32] concluded that PNs were knowledgeable. An Australian national survey confirmed the generalist role with PNs seeing patients who have a wide range of chronic conditions [47]. However, there is clearly a tension among PNs and GPs to remain generalists and pressure to become primary care specialists in care of people with diabetes or coronary heart disease (CHD) [56]. Provision of simple lifestyle information and advice was predominant strategy used by PNs [53]. PNs self-reported they would benefit from further training about nutrition knowledge and obesity [36]. In the survey of Hankey et al. (2003) [46] both PNs and GPs felt that dieticians should hold specialist posts in weight management.

Moreover, nine studies dealt with competency issues. PNs were rather ambiguous about their role [43,51]. Furthermore, both PNs and GPs did not agree on the PNs’ role [12,16,44,46,49] or showed only some congruence in opinion [37]. Australian PNs felt competent at providing basic nutrition care [50]. However, PNs had better interpersonal skills than GPs [16]. The review of Horrocks et al. (2002) [57] showed that NPs may have superior interpersonal skills than doctors.

With respect to type of intervention activity, the majority of studies reported on lifestyle counselling, while only eight included weight management [20,22,23,36,38,39,45,46].

## Conclusion

Our review about PNs’ actual role in lifestyle counselling in primary care reveals that we might be just at the beginning of understanding how PNs can contribute to lifestyle counselling or how PNs best fit into the primary health care team. A variety of studies have been found, ranging from differences in self-reported roles between PNs and GPs to intervention studies and observations of real-life counselling. We will elaborate on four main outcomes.

First, in some countries, such as the UK, the Netherlands, and Scandinavian countries, PNs can play the role of case manager lifestyle counselling regarding weight management in primary care. They are supervised by GPs, within primary health care team different roles are considered as clear, and cooperation is going well. Lifestyle counselling regarding weight management can be seen as the first step in the right direction in order to manage even more cases. In countries in which a primary health care team is still in development and there is no agreement on respective roles, such as the USA, it is still the question whether the PN can play the case manager role, because they need to agree on their roles in primary health care team and feel this teamwork is feasible, attractive and satisfactory.

Moreover, PNs’ role in chronic disease management is spreading increasingly into lifestyle counselling. Already in 2001, Katon et al. [58] suggested that GPs diagnose and initiate treatment and lifestyle counselling, whereas PNs monitor treatment outcome, provide counselling and support for behavior change, and offer follow-contacts. Patients themselves were generally satisfied with PNs’ lifestyle counselling [59,60], and perceived that PN have more available time [61,62].

Furthermore, we found that PNs experience the same barriers for lifestyle counselling as GPs. PNs have more time than GPs to provide lifestyle counselling, and frequency increased in recent years. Nevertheless, lack of time was still an important barrier. The review of Tulloch et al. (2006) [63] including 19 studies suggested that non-physician providers may be better suited for providing physical activity counselling due to an ability to provide a more intensive intervention. Moreover, the review of Fokkens et al. (2011) [64] including 10 studies indicated that interventions in which the nurse fulfils the role of the primary care provider result in larger effects on clinical outcomes.

Finally, a variety of PNs’ competences are required for effective lifestyle counselling. The review of Smith (2011) [65] including 11 studies found that nurses can expect to experience substantial role ambiguity and role conflict. In the Counterweight Project Team (2004) [39] for example, PNs were trained dieticians, who received competence training with respect to communication skills and behavioral change.

A strength of this study is the systematic way in which an electronic literature search was performed in order to provide state of the art on main outcomes of studies about PNs’ actual role in lifestyle counselling in primary care and cooperation with other health professionals.

A possible limitation is the grey area with (yet?) unpublished literature. Moreover, we found a lot of studies published after 2010, so some studies might still be in the pipeline. Unfortunately, we did not have access to the CINAHL database, so we might miss some specific nursing papers. Definition and education level of PNs differed between countries, as well as the primary health care systems and variety of variables, resulting in a heterogeneous mix of studies. Furthermore, the quality of studies was not very high. Due to the low number of (national) representative studies, caution is warranted for making comparisons.

### Practice implications

Further training is required before case management of chronic diseases in primary care becomes an integral part of the role of PNs in Ireland [37]. The majority of American NPs are interested in receiving additional training to aid in providing physical activity counselling [32]. Competency training in both general communication skills and motivational interviewing skills are recommended.

According to Frank (1998) [66], shifting the major responsibility to non-physician professionals may offer the most promising therapeutic opportunities for obesity management. A review including 11 studies showed that there is potential in primary care nursing to help patients manage obesity [67]. Recently, a study suggested that PNs are well placed to perform two key roles in obesity management, namely counselling patients who have obesity-related co-morbidities and identification of patients who are overweight and healthy [68]. Recently, an observational study identified that PNs’ goal setting could be improved [69]. The quality of PNs' weight loss counselling might be increased by routinely providing assistance in addressing barriers and securing support, and routinely reaching agreement with collaboratively set goals [70]. A multidisciplinary team approach to weight management is preferable, with training of PNs and specialized dieticians to address this issue [46]. Closer liaison with dietetic services could allow dietician expertise regarding weight management to be utilized more fully [36].

It seems to be crucial that members of the primary health care team agree on their respective roles, and feel this teamwork is feasible, attractive, and satisfactory. Monitoring these roles with quantitative studies is recommended.

In conclusion, because of the heterogeneity of the studies, the lack of (national) representative studies and the differences between countries (among other factors), there is no definite answer to the question whether or not the PN can act as case manager lifestyle counselling regarding weight management in primary care. It depends on the context of the situation and the country.

## Additional file

Additional file 1:
**Main characteristics, main outcomes and study quality of studies about PNs’ involvement in lifestyle counselling [** 71,72 **].**

## Abbreviations

PN: Practice nurse
GP: General practitioner
RCT: Randomized controlled trial
NP: Nurse practitioner
CHD: Coronary heart disease

## References

[1] Heiligers PJM, Noordman J, Korevaar JC, Dorsman S, Hingstman L, Van Dulmen AM, De Bakker DH (2012). Praktijkondersteuners in de huisartspraktijk (POH’s), klaar voor de toekomst? [Practice nurses in general practice, ready for the future?].

[2] Macdonald W, Rogers A, Blakeman T, Bower P (2008). Practice nurses and the facilitation of self-management in primary care. J Adv Nurs.

[3] Bourgueil Y, Marek A, Mousquès J (2005). The participation of nurses in primary care in six European countries, Ontario and Quebec. Health Economics Letter, no. 95.

[4] Van Dillen SME, Van Binsbergen JJ, Koelen MA, Hiddink GJ (2013). Nutrition and physical activity guidance practices in general practice: a critical review. Patient Educ Couns.

[5] Hiddink GJ, Hautvast JGAJ, Van Woerkum CMJ, Fieren CJ, Van ‘t Hof MA (1997). Driving forces for and barriers to nutrition guidance practices of Dutch primary care physicians. J Nutr Educ.

[6] Fransen GAJ, Hiddink GJ, Koelen MA, Van Dis SJ, Drenthen AJM, Van Binsbergen JJ, Van Woerkum CMJ (2008). The development of a minimal intervention strategy to address overweight and obesity in adult primary care patients in The Netherlands. Fam Pract.

[7] Sargent GM, Forrest LE, Parker RM (2012). Nurse delivered lifestyle interventions in primary health care to treat chronic disease risk factors associated with obesity: a systematic review. Obes Rev.

[8] Laurant M, Reeves D, Hermens R, Braspenning J, Grol R, Sibbald B (2004). Substitution of doctors by nurses in primary care. Cochrane Database Syst Rev.

[9] Swinburn BA, Sacks G, Hall KD, McPherson K, Finegood DT, Moodie ML, Gortmaker SL (2011). The global obesity pandemic: shaped by global drivers and local environments. Lancet.

[10] National Institute on Health and Clinical Excellence (NICE) (2006). Obesity: the prevention, identification, assessment and management of overweight and obesity in adults and children.

[11] Whitlock EP, Orleans T, Pender N, Allan J (2002). Evaluating primary care behavioural counselling interventions: an evidence-based approach. Am J Prev Med.

[12] Donelan K, DesRoches CM, Dittus RS, Buerhaus P (2013). Perspectives of physicians and nurse practitioners on primary care practice. New Engl J Med.

[13] Case Management Society of America (CMSA): **What is a case manager?** [http://www.cmsa.org/Consumer/FindaCaseManager/WhatisaCaseManager/tabid/276/Default.aspx]

[14] Helmink JH, Meis JJ, De Weerdt I, Visser FN, De Vries NK, Kremers SPJ (2010). Development and implementation of a lifestyle intervention to promote physical activity and healthy diet in the Dutch general practice setting: the BeweegKuur programme. Int J Behav Nutr Phys Act.

[15] Duijzer G, Jansen SC, Haveman-Nies A, Van Bruggen R, Ter Beek J, Hiddink GJ, Feskens EJM (2012). Translating the SLIM diabetes prevention intervention into SLIMMER: implications for the Dutch primary health care. Fam Pract.

[16] Philips CB, Pearce C, Hall S, Kljakovic M, Sibbald B, Dwan F, Porritt J, Yates R (2009). Enhancing care, improving quality: the six roles of the general practice nurse. Med J Aus.

[17] Marsh GN, Dawes ML (2000). Establishing a minor illness nurse in a busy general practice. BMJ.

[18] Burns KJ, Camaione DN, Chatterton CT (2000). Prescription of physical activity by adult nurse practitioners: a national survey. Nurs Outlook.

[19] Campbell NC, Thain J, Deans HG, Ritchie LD, Rawles JM, Squair JL (1998). Secondary prevention clinics for coronary heart disease: randomized trial of effect on health. BMJ.

[20] Ter Bogt NCW, Bemelmans WJE, Beltman FW, Broer J, Smit AJ, Van der Meer K (2009). Preventing weight gain: one-year results of a randomized lifestyle intervention. Am J Prev Med.

[21] Koelewijn-Van Loon MS, Van der Weijden T, Ronda G, Van Steenkiste B, Winkens B, Elwyn G, Grol R (2010). Improving lifestyle and risk perception through patient involvement in nurse-led risk management: a cluster-randomized controlled trial in primary care. Prev Med.

[22] Vermunt PWA, Milder IEJ, Wielaard F, De Vries JHM, Van Oers HAM, Westert GP (2011). Lifestyle counselling for type 2 diabetes risk reduction in Dutch primary care: results of the APHRODITE study after 0.5 and 1.5 years. Diab Care.

[23] Voogdt-Pruis HR, Van Ree JW, Gorgels APM, Beusmans GHMI (2011). Adherence to a guideline on cardiovascular prevention: A comparison between general practitioners and practice nurses. Int J Nurs Stud.

[24] Driehuis F, Barte JCM, Ter Bogt N, Smit AJ, Van der Meer K, Bemelmans WJE (2012). Maintenance of lifestyle changes: 3-year results of the Groningen Overweight and Lifestyle study. Patient Educ Couns.

[25] Little P, Dorward M, Gralton S, Hammerton L, Pillinger J, White P, Moore M, McKenna J, Payne S (2004). A randomized controlled trial of three pragmatic approaches to initiate increased physical activity in sedentary patients with risk factors for cardiovascular disease. Br J Gen Pract.

[26] Kinnersley P, Anderson E, Parry K, Clement J, Archard L, Turton P, Stainthorpe A, Fraser A, Butler CC, Rogers C (2000). Randomised controlled trial of nurse practitioner versus general practitioner care for patients requesting “same day” consultations in primary care. BMJ.

[27] Shum C, Humphreys A, Wheeler D, Cochrane MA, Skoda S, Clement S (2000). Nurse management of patient with minor illnesses in general practice: multicentre, randomised controlled trial. BMJ.

[28] Venning P, Durie A, Roland M (2000). Randomised controlled trial comparing cost effectiveness of general practitioners and nurse practitioners in primary care. BMJ.

[29] McDowell N, McKenna J, Naylor PJ (1997). Factors that influence practice nurses to promote physical activity. Br J Sports Med.

[30] Douglas F, Torrance N, Van T, Meloni S, Kerr A (2006). Primary care staff’s views and experiences related to routinely advising patients about physical activity. A questionnaire survey. BMC Pub Health.

[31] Buchholz SW, Purath J (2007). Physical activity and physical fitness counselling patterns of adult nurse practitioners. J Am Acad Nurs Pract.

[32] Grimstvedt ME, Der Ananian C, Keller, Woolf K, Sebren A, Ainsworth B (2012). Nurse practitioner and physician assistant physical counseling knowledge, confidence and practices. Prev Med.

[33] Poskiparta M, Kasila K, Kiuru P (2006). Dietary and physical activity counselling on Type 2 diabetes and impaired glucose tolerance by physicians and nurses in primary healthcare in Finland. Scand J Prim Health Care.

[34] Noordman J, Koopmans B, Korevaar JC, Van der Weijden T, Van Dulmen S (2013). Exploring lifestyle counselling in routine primary care consultations: the professionals’ role. Fam Pract.

[35] Steptoe A, Doherty S, Kendrick T, Rink E, Hilton S (1999). Attitudes to cardiovascular health promotion among GPs and practice nurses. Fam Pract.

[36] Green SM, McCoubrie M, Cullingham C (2000). Practice nurses’ and health visitors’ knowledge of obesity assessment and management. J Hum Nutr Diet.

[37] McCarthy G, Cornally N, Moran J, Courtney M (2012). Practice nurses and general practitioners: perspectives on the role and future development of practice nursing in Ireland. J Clin Nurs.

[38] Michie S (2007). Talking to primary care patients about weight: A study of GPs and practice nurses in the UK. Psych Health Med.

[39] The Counterweight Project Team (2004). Current approaches to obesity management in UK Primary Care: the Counterweight Programme. J Hum Nutr Diet.

[40] Hernandez J, Anderson S (2012). Storied experiences of nurse practitioners managing prehypertension in primary care. J Am Acad Nurs Pract.

[41] Laurant MGH, Hermens RPMG, Braspenning JCC, Sibbald B, Grol RPTM (2004). Impact of nurse practitioners on workload of general practitioners: randomised controlled trial. BMJ.

[42] Moher M, Yudkin P, Wright L, Turner R, Fuller A, Schofield T, Mant D (2001). Cluster randomized controlled trial to compare three methods of promoting secondary prevention of coronary heart disease in primary care. BMJ.

[43] Halcomb EJ, Davidson PM, Salamonson Y, Ollerton R, Griffiths R (2008). Nurses in Australian general practice: implications for chronic disease management. J Clin Nurs.

[44] Patterson E, Del Mar C, Najman C (1999). Nursing’s contribution to general practice: general practitioners’ and practice nurses’ views. Collegian.

[45] Hoppé R, Ogden J (1997). Practice nurses’ beliefs about obesity and weight related interventions in primary care. Int J Obes.

[46] Hankey CR, Eley S, Leslie WS, Hunter CM, Lean MEJ (2003). Eating habits, beliefs, attitudes and knowledge among health professionals regarding the links between obesity, nutrition, and health. Pub Health Nutr.

[47] Joyce CM, Piterman L (2011). The work of nurses in Australian general practice: A national survey. Int J Nurs Stud.

[48] Bailey P, Jones L, Way D (2006). Family practitioner/nurse practitioner: stories of collaboration. J Adv Nurs.

[49] Geense WW, Van de Glind IM, Visscher TLS, Van Achterberg T (2013). Barriers, facilitators and attitudes influencing health promotion activities in general practice: an explorative pilot study. BMC Fam Pract.

[50] Cass S, Ball L, Leveritt M (2013). Australian practice nurses’ perceptions of their role and competency to provide nutrition care to patients living with chronic disease. Aus J Prim Health.

[51] McDonald R, Campbell S, Lester H (2009). Practice nurses and the effects of the new general practitioners contract in the English National Health Service: The extension of a professional project?. Soc Sci Med.

[52] Lambe B, Collins C (2010). A qualitative study of lifestyle counselling in general practice in Ireland. Fam Pract.

[53] Jansink R, Braspenning J, Van der Weijden T, Elwyn G, Grol R (2010). Primary care nurses struggle with lifestyle counseling in diabetes care: a qualitative analysis. BMC Fam Pract.

[54] Mitchell LJ, MacDonald-Wicks L, Capra S (2011). Nutrition advice in general practice: the role of general practitioners and practice nurses. Aus J Prim Health.

[55] Helmink JHM, Kremers SPJ, Van Boekel LC, Van Brussel-Visser FN, De Vries NK (2012). Factors determining the motivation of primary health care professionals to implement and continue the ‘Beweegkuur’ lifestyle intervention programme. J Eval Clin Pract.

[56] Williams R, Rapport F, Elwyn G, Lloyd B, Rance J, Belcher S (2004). The prevention of type 2 diabetes: general practitioner and practice nurse opinions. Br J Gen Pract.

[57] Horrocks S, Anderson E, Salisbury C (2002). Systematic review of whether nurse practitioners working in primary care can provide equivalent care to doctors. BMJ.

[58] Katon W, Von Korff M, Lin E, Simon G (2001). Rethinking practitioner roles in chronic illness: the specialist, primary care physician, and the practice nurse. Gen Hosp Psych.

[59] Duaso MJ, Cheung P (2002). Health promotion and lifestyle advice in a general practice: what do patients think?. J Adv Nurs.

[60] Wilson PM, Brooks F, Procter S, Kendall S (2012). The nursing contribution to chronic disease management: A case of public expectation? Qualitative findings from a multiple case study design in England and Wales. Int J Nurs Stud.

[61] Hayes E (2007). Nurse practitioners and managed care: Patient satisfaction and intention to adhere to nurse practitioner plan of care. J Am Acad Nurs Pract.

[62] Mahomed R, St John W, Patterson E (2012). Understanding the process of patient satisfaction with nurse-led chronic disease management in general practice. J Adv Nurs.

[63] Tulloch H, Fortier M, Hogg W (2006). Physical activity counselling in primary care: Who has and who should be counselling?. Patient Educ Couns.

[64] Fokkens AS, Wiegersma PA, Reijneveld SA (2011). Organization of diabetes primary care: a review of interventions that delegate general practitioner tasks to a nurse. J Eval Clin Pract.

[65] Smith AC (2011). Role ambiguity and role conflict in nurse case managers: an integrative review. Prof Case Man.

[66] Frank A (1998). A multidisciplinary approach to obesity management: the physician’s role and team care alternatives. J Am Diet Ass.

[67] Brown I, Psarou A (2007). Literature review of nursing practice in managing obesity in primary care: developments in the UK. J Clin Nurs.

[68] Philips K, Wood F, Kinnersley P (2014). Tackling obesity: the challenge of obesity management for practice nurses in primary care. Fam Pract.

[69] Van Dillen SME, Noordman J, Van Dulmen S, Hiddink GJ (2014). Examining the content of weight, nutrition and physical activity advices by Dutch practice nurses in primary care: analysis of video-taped consultations. Eur J Clin Nutr.

[70] Van Dillen SME, Noordman J, Van Dulmen S, Hiddink GJ: **Quality of weight-loss counseling by Dutch practice nurses in primary care: an observational study.** *Eur J Clin Nutr* 2014. doi:10.1038/ejcn.2014.129. 10.1038/ejcn.2014.129 24986823

[71] Kiuru P, Poskiparta M, Kettunen T, Saltevo J, Liimatainen L (2004). Advice-giving styles by Finnish nurses in dietary counselling concerning type 2 diabetes care. J Health Com.

[72] Goetz K, Szecsenyi J, Campbell S, Rosemann T, Rueter G, Raum E, Brenner H, Miksch A (2012). The importance of social support for people with type 2 diabetes – a qualitative study with general practitioners, practice nurses and patients. GMS Psychosoc Med.

