# Supplementary material for: To what extent do primary care practice nurses act as case managers lifestyle counselling regarding weight management? A systematic review
Source: BMC Fam Pract. 2014 Dec 10;15:197. doi: 10.1186/s12875-014-0197-2 (PMC4269898; doi:10.1186/s12875-014-0197-2)
Supplement: Additional file 1: — Main characteristics, main outcomes and study quality of studies about PNs’ involvement in lifestyle counselling [ 71,72 ]. [file 12875_2014_197_MOESM1_ESM.pdf]

Additional file 1: Main characteristics, main outcomes and study quality of studies about PNs' involvement in lifestyle counselling

Author, year, country	Study design, sample	Main outcomes	Study quality
Marsh & Dawes [17], 1995, USA	One group practice, 1 nurse, 696 consultations	86% of consultations required no doctor contact; 50% were given advice on self-care	Low
Hoppé & Ogden [45], 1997, UK	Cross-sectional questionnaire, random sample, 586 PNs	The majority gave advice more than once a week but most spend 10 minutes or less discussing weight loss; they reported offering general nutrition advice and exercise most frequently; eat less in general and referrals to a self-help group were offered sometimes, and calorie controlled diets least frequently	Medium
McDowell et al. [29], 1997, UK	Postal questionnaire, all PNs in 1 region,220 PNs	Over 80% currently promotes physical activity; most new patients were asked about their activity habits; verbal advice was most common, followed by giving out pamphlets	Medium
Campbell et al.[19], 1998, UK	RCT, random sample of 19 general practices in 1 region, intervention group secondary prevention clinic run by	Significant improvements in 6 of 8 health status domains were made (physical functioning/social functioning/role limitations attributed to physical problems/pain/general health perception); fewer patients reported worsening chest pain; fewer patients required hospital admissions, but GP consultation rates did not	High

	nurses (593 patients) vs. control group (580 patients)	alter	
Patterson et al. [44], 1999, Australia	Mailed survey, selected sample within 1 division of General Practice, 37 PNs and 84 GPs	Both PNs and GPs agreed that PNs' contribution to preventive health care was underutilized (80% vs. 70%); 77% of PNs agreed that PNs should carry out planned health promotion, while 46% of GPs agreed	Medium
Steptoe et al. [35], 1999, UK	Questionnaire survey, sample of 58 PNs and 107 GPs	Both PNs and GPs agreed that PNs are most appropriate to carry out health promotion (64% vs. 59%); significantly more PNs than GPs agreed that their job was to act as health educator and to treat disease; majority of PNs disagreed they had no time for preventive medicine; more PNs considered work included detection of obesity or physical activity	Medium
Burns et al. [18], 2000, USA	National survey, mail questionnaire, random sample, 606 NPs	58% reported providing routine physical activity counselling to patients; 99% discussed physical activity; 67% provided written information; 43% referred to exercise specialist; the most recommended activities were walking (98%), swimming (70%), biking (59%) and household activities (51%); 74% advised a frequency of 3 times a week; 66% advised moderate intensity, such as brisk walk; 49% advised a talk test; 46% advised to exercise at 60-85% of maximum heart rate	High

Green et al. [36], 2000; UK	Postal questionnaire, selection of 24 practices within 1 regional health authority, 28 PNs (and 17 health visitors)	All PNs provided dietary and lifestyle advice to obese patients; a low-fat diet was most frequently recommended; all PNs advised obese patients to include physical activity in their lifestyle; walking was most often recommended	Medium
Kinnersley et al. [26], 2000, UK	RCT, 10 general practices, NP group (652 patients seeking "same day consultations") and GP group (716 patients)	Patients consulting NPs were significantly more satisfied with their care, received significantly more information about their illnesses, and their consultations were significantly longer	High
Venning et al. [28], 2000, UK	RCT, 20 general practices, NP group (641 patients) vs. GP group (651 patients)	NP consultations were significantly longer than those of GPs (12 resp. 7 minutes); NPs carried out more tests (opportunistic screening)(9 resp. 6%) and asked patients to return more often (37 resp. 25%); patients were more satisfied with NP consultations	Medium
Shum et al. [27], 2000, UK	RCT, 5 general practices in 2 cities, nurse group (888 patients) vs. GP group (904 patients)	Patients were significantly more satisfied with their consultations with nurses; consultations with nurses took about 10 minutes compared with about 8 minutes with GPs; 73% of patients seen by nurses were managed without any input from GPs	High

Moher et al. [42], 2001, UK	RCT, 21 general practices, nurse group (682 patients) vs. GP group (682 patients) vs. audit group (559 patients)	Adequate assessment of 3 risk factors for CHD (blood pressure, cholesterol, and smoking status) was much more common in the nurse and GP groups (85 resp. 76%) than the audit group (52%); differences in assessment were not reflected in clinical outcomes	High
Hankey et al. [46], 2003, UK	Self-complete questionnaire postal survey, stratified sample, 509 PNs, 741 GPs (and 244 dieticians)	86% of PNs agreed that nurses should provide weight-reduction diets against 78% of GPs; 73% of PNs agreed that GPs should offer advice to overweight patients against 66% of GPs; 76% agreed that there should be specialist posts for dieticians in weight management against 66% of GPs; only 10% audited patients records to identify BMI	Medium
Kiuru et al. [71], 2004, Finland	Observational study, collection of all subsequent meetings of 18 diabetes patients in 4 primary care centres, 55 video-taped sessions between 18 voluntary patients with diabetes and 5 nurses	Recommending style was present in 52% of speech episodes, followed by permitting (28%), supportive (28%) and persuasive style (7%)	Low

Laurant et al. [41], 2004, the Netherlands	RCT, selection of 34 general practices in 1 region, intervention group (5 NPs) vs. control group (no nurse)	The number of contacts during surgery hours increased in the intervention group compared with control group; the number of consultations out of hours declined, but not significantly; no significant changes in subjective workload were found	High
Little et al., 2004 [25], UK	RCT, selection from 4 practice settings, 7 interventions (nurse; nurse/booklet; nurse/ GP; GP/ nurse/booklet; GP; GP/booklet; booklet) and control group, 151 patients, number of GPs and PNs unknown	Only with the most intensive intervention (GPs' exercise prescription and PNs' counselling combined) there were significant increases in physical activity and fitness	Medium
The Counterweight Project Team, 2004 [39], UK	Structured interviews, selection of 40 practices from those reacting positively, 66 PNs and 141 GPs; audit of	76% of PNs reported discussing weight-related issues against 15% of GPs; PNs also more often reported that they would raise weight as an issue, set personal lifestyle goals, provide individualized dietary prescription; 78% of GPs referred to PN	Medium

	100 obese patients' records		
Williams et al. [56], 2004, UK	Qualitative study, focus groups, approach, approaching all practices in 1 local health board area, 22 PNs and 21 GPs	PNs and GPs hold pessimistic views of activities related to detection of those at high risk for diabetes, coupled with cynicism about successful behavioural change, and putting responsibility on other sectors	Low
Bailey et al. [48], 2006, Canada	Interviews, 4 rural primary care practices, 5 NPs and 13 family physicians	Identified themes were scope of practice, emphasizing the importance of role clarity and trust, the ideological difference regarding disease prevention and health promotion, differences in perceptions about the operation of collaborative practices, and the understanding that collaborative relationships evolve	Low
Douglas et al. [30], 2006, UK	Questionnaire survey, postal questionnaire and 20 in-depth interviews, 4 health regions, 186 PNs (and 149 health visitors)	88% of PNs said they were (very) likely to recommend all healthy patients to take moderate exercise; walking was most recommended; the majority tailored their advice	High
Poskiparta et al. [33], 2006,	Observational study, selection of 4 primary care	Dietary counselling was present in 79% of sessions with nurses against 63% of physicians; physical activity counselling occurred in 77% of sessions with nurses	Low

Finland	organizations, 129 video-taped counselling sessions of 17 patients with 5 nurses and 7 physicians	against 50% of physicians	
Buchholz & Purath [31], 2007, USA	Questionnaire, random sample, 96 NPs	74% reported providing routine physical activity to patients; 95% discussed physical activity; 54% gave written materials; 57% advised to engage in physical activity most days of the week; 66% advised this for 30 minutes; 65% compared the activity to the intensity of a brisk walk; 55% used the talk test, and 38% the target heart rate of 60-85% of maximum heart rate	High
Michie [38], 2007, UK	Cross-sectional study, selection of 2 urban primary care organisations, postal questionnaire, 47 PNs and 40 GPs	PNs were more likely to raise the issue of weight than GPs, both opportunistically and when medical problem; only 9% presented solutions to weight loss or discussed health promotion	Medium
Halcomb et al. [43], 2008, Australia	National postal survey, convenience sample, 284 PNs	There was a mismatch between the appropriateness and undertaking the task; legal and funding issues, lack of space, and GP attitudes were identified as barriers	Medium

Macdonald et al. [2], 2008, UK	Semi-structured interviews, convenience sample, 25 PNs	Main themes in the early stages were categorization of patients, diagnosis, and patient education; intermediate stages were 'ways of working' and maintaining relationships with patients; in the later stages they lack resources beyond personal experience and intuitive ways for self-care.	High
McDonald et al. [51], 2009, UK	Interviews, sample from a national-representative sample of 43 practices, 20 PNs	PN work is changing to reflect a more medical orientation, while nursing work is described as routine and template driven, which may limit claims to professional status; they took on greater responsibility for the management of chronic disease	Low
Philips et al. [16], 2009, Australia	Cross-sectional and longitudinal study, multi-method, selection of 25 practices in two regions, interviews with 36 nurses, 24 doctors, and 22 practice managers, observations of 34 nurses, action research	Both nurses and doctors appreciated 3 nurse' roles as nursing strengths, namely patient carer, organiser, and quality controller; doctors tended not to recognise nurses' educator and problem solvers roles within the practice; the role as agent of connectivity is particularly notable in small and medium practices; nurses high lightened the personal importance of positive patient interactions	Low
Ter Bogt et al.	RCT, questionnaire, random	There were more weight losers and stabilizers in the NP group than in GP group	High

[20], 2009, the Netherlands	sample, NP intervention including lifestyle counselling (225 patients) vs. GP usual care (232 patients), unknown number of NPs and GPs	(77% vs. 65%); mean weight losses in men were 2% for NP group vs. 0.1 % for GP group; obese people in NP group lost more weight than non-obese (-3% vs. -1%)	
Jansink et al. [53], 2010, the Netherlands	Semi-structured in-depth interviews, selection of the first 12 of 70 practices participating in a RCT, 12 primary care nurses	Nurses felt most barriers on the level of the patient: limited knowledge and lack of motivation; they also reported lack of counselling skills and insufficient time as barriers	Medium
Koelewijn-Van Loon et al. [21], 2010, the Netherlands	RCT, questionnaire, cluster random sample, PN intervention including motivational interviewing (13 PNs, 320 patients eligible for cardiovascular risk management) vs. PN usual	Both patients of intervention and usual care group improved their lifestyle, but not significant; intervention group patients improved risk perception, anxiety an satisfaction with the communication	High

	care (11 PNs, 293 patients)		
Lambe & Collins [52], 2010, Ireland	Focus groups, purposeful sample in urban and rural locations, 56 participants (PNs and GPs)	PNs and GPs experienced considerable barriers to lifestyle counselling, including patient resistance, lack of time, funding and training; provision of simple lifestyle advice was the predominant strategy used	Medium
Joyce & Piterman [47], 2011, Australia	National cross-sectional survey, convenience sample, 104 GP nurses, 5253 nurse-patient encounters	GP nurses are involved in screening and preventive activities as well as monitoring and management of chronic conditions; 30% of encounters involved advice-giving	Medium
Mitchell et al. [54], 2011, Australia	Mixed methods, questionnaires among 12 PNs, 10 GPs, and 13 patients (and semi structured telephone interviews among 52 dieticians and online survey among 90 dieticians)	The provision of basic nutrition advice is acknowledged to be part of the role of GPs and practice nurses; PNs reported spending more time discussing nutrition than GPs(5-10 minutes vs. 1-5 minutes)	Low
Vermunt et al.	RCT, questionnaire, selection	Significant changes were found for total physical activity, energy intake, and fibre	Medium

[22], 2011, the Netherlands	of 14 primary care practices, lifestyle intervention (NPs and GPs, 330 patients) vs. usual care group (NPs and GPs, 305 patients), unknown number of NPs and GPs	intake; NPs' mean year of work experience was longer in patients who were successful at losing weight or maintaining a stable weight compared to the unsuccessful	
Voogdt-Pruis, et al. [23], 2011, the Netherlands	RCT, cross-sectional questionnaire survey, random sample, PN intervention (6 PNs, 314 high risk patients) vs. GP intervention (25 GPs, 387 high risk patients)	More lifestyle intervention was given by the PN: 46% of patients received at least 1 lifestyle intervention (weight, diet, exercise, and smoking) compared to 13% in GP group	High
Driehuis et al. [24], 2012, the Netherlands	RCT, questionnaire, random sample, NP intervention including lifestyle counselling (225 patients) vs. GP usual care (232 patients), unknown	After 3 years, leisure-time physical activity increased and favourable improvements towards a healthy diet were made for GP and NP group	Medium

	number of NPs and GPs		
Goetz et al. [72], 2012, Germany	Qualitative study, focus groups and interviews, recruitment from quality circle in 1 area, 10 PNs, 10 GPs (and 10 diabetes patients)	PNs and GPs advised patients on nutrition and physical activity; most PNs reported their role in the provision of social support, regular and on-going care, and invitation of family members; GPs emphasized PNs' role in support	Low
Grimstvedt et al. [32], 2012, USA	Cross-sectional survey, online sample or in person during 2 conferences, 240 NPs (and 78 physician assistants)	75% reported routinely counselling patients about physical activity; 90% indicated that discussing physical activity with patients was their primary counselling method and were most likely to recommend physical activity on 3 days per week (43%), 50% were more likely to recommend the "talk test" as their primary recommended measure of intensity for patients, walking was the most prescribed activity (90%)	Medium
Helmink et al. [55], 2012, the Netherlands	Questionnaire, selection of primary health care providers who took part in a pilot intervention scheme, 12 PNs, 7 GPs (and 16 physiotherapists)	The motivation of PNs to continue implementation was lower compared with other professionals	Medium

Hernandez & Anderson [40], 2012, USA	Interviews, purposive sample, 8 NPs caring for pre-hypertensive patients	Three themes were identified: realities of practice, ambiguous role identity, and bridging models; barriers identified were time constraints, financial considerations and bridging models	Low
McCarthy et al. [37], 2012, Ireland	Cross-sectional survey, purposeful sample of PNs and random sample of GPs, 451 PNs and 414 GPs	PNs and GPs agree that the nursing role is centred on elements of chronic disease management; PNs were more likely to indicate competency in health promotion activities	Medium
Cass et al. [50], 2013, Australia	Qualitative study, semi-structured interview, purposive sampling, 20 PNs	PNs perceived that the ideal role is to advocate for nutrition and provide a basic level of nutrition care to patients; barriers included time constraints, lack of knowledge, and lack of confidence	Low
Donelan et al. [12], 2013, UK	National postal-mail survey, random samples, 467 NPs and 505 PCPs	NPs were more likely to believe than PCPs that they should lead medical homes, be allowed hospital admitting privileges, and be paid equally for the same clinical services; 28% of PCPs agreed that NPs provided services for complex chronic conditions that were complicated by coexisting conditions or were not yet well controlled (vs. 68% of NPs)	High
Geense et al.	Semi-structured interviews,	PNs reported they give lifestyle counselling to chronically ill patients, actively ask	Low

[49], 2013, the Netherlands	purposive sample, 9 PNs and 16 GPs	about lifestyle, give lifestyle advice, use motivational interviewing, and refer to other disciplines; GPs also discussed lifestyle, those who are ‘confirmers’ emphasized the importance of PNs; the most cited facilitator is availability of a PN	
Noordman et al. [34], 2013, the Netherlands	Observational study, GP sample from the Netherlands information network of general practice, GPs and GPs from another practice were contacted for participation of PNs, 19 PNs and 39 GPs, 124 and 141 patients	PNs more often discussed physical activity than GPs (84% vs. 61%); PNs more often discussed nutrition than GPs (80% vs. 43%)	Medium
